# Suitable ultrasound screening method for older adults with disability to identify low muscle mass

**DOI:** 10.3389/fmed.2023.1270176

**Published:** 2023-10-05

**Authors:** Huaying Ding, Xia Lin, Sha Huang, Jie Liao, Zhouyu Li, Lanlan Chen, Li Zhu, Yukuan Xie, Qian Nie, Xiaoyan Chen

**Affiliations:** Zigong Psychiatric Research Center, Zigong Affiliated Hospital of Southwest Medical University, Zigong, Sichuan Province, China

**Keywords:** ultrasound, low muscle mass, measurement scheme, older, accuracy, consistency

## Abstract

**Objective:**

This study aimed to investigate the accuracy and consistency of different ultrasound protocols for the measurement of gastrocnemius muscle (GM) thickness and to identify a suitable ultrasound scheme that can be used to detect the low muscle mass in older with disability.

**Materials and methods:**

In this cross-sectional study, each participant underwent three different ultrasound protocols for the measurement of the GM thickness, and each measurement was repeated three times. The three measurement schemes were as follows: method A, lying on the examination bed in a prone position with legs stretched and relaxed and feet hanging outside the examination bed; method B, lateral right side lying position with legs separated (left leg flexed and right leg in a relaxed state); and method C, right side lying position with legs together and lower limb muscles in a relaxed state. The low muscle mass was determined by averaging two or three measurements of the GM thickness determined using different sonographic protocols.

**Results:**

The study included 489 participants. The difference in the prevalence of low muscle mass identified between two and three replicates of the same measurement protocol ranged from 0 to 1.3%. Considering the three repeated measurements of the method A as the reference, the area under the curve (AUC) in different measurement schemes were 0.977-1 and 0.973-1 in males and females, respectively. Furthermore, male and female Kappa values from low to high were 0.773, 0.801, 0.829, 0.839, and 0.967 and 0.786, 0.794, 0.804, 0.819, and 0.984, respectively.

**Conclusion:**

Different ultrasound measurement protocols showed high accuracy and consistency in identifying low muscle mass. Repeating the measurements two or three times was found to be feasible.

## Introduction

Disability refers to impairments, limitations in activity, and restricted participation (1, 2). The prevalence of disability among middle– and old-age individuals ranges from 19.4 to 33.3% (1–5), and it is positively correlated with age (5, 6). Disability significantly increases the rehospitalization risk and frailty, decreases quality of life, and can even lead to death (3, 7, 8). In comparison to older adults without disability, those with disability spend approximately $10,000 more in 2 years (9). Disability and sarcopenia are closely related; sarcopenia is one of the important causes of disability (10–12). Individuals with disability are prone to sarcopenia due to limited activities (13). Hence, people with disability and sarcopenia experience more personal, social, and economic burdens.

Sarcopenia is defined as loss of muscle mass, which is age-related, and it is also characterized by low muscle strength and/or low physical performance (14). Low muscle mass is an important element in the sarcopenia diagnosis (14). At present, common methods used for the measurement of muscle mass include bioelectrical impedance analysis (BIA) and dual energy X-ray absorption (DEXA) (14). However, the application of these two methods in individuals with disability is difficult. For instance, during the BIA, knee extension is required, but in a few people with disability, the knee joints are stiff and flexed, restricting extension. Furthermore, BIA is required to be performed empty stomach, which may not be possible for a few patients with disability. On the other hand, DEXA is a method in which radiation is used, and this method lacks portability and is inconvenient for older adults with disability and limited activity. Considering these shortcomings of available methods for measuring the muscle mass, the identification of an alternative method is necessary.

Ultrasonic measurement of the muscle mass is associated with advantages, such as no requirement of knee joint extension or flexion, no diet effect, no use of radiation, and portability. Multiple studies have demonstrated reliable performance of ultrasound method in assessing the muscle mass (15–18). A meta-analysis reported the area under the summary receiver operating characteristic curve [SROC] of 0.76 for ultrasonic measurement of gastrocnemius muscle (GM) thickness to diagnose low muscle mass, and its correlation with DEXA, which is a gold-standard method for diagnosing low muscle mass, was also high (*r* = 0.85–0.963) (19). GM thickness is an appropriate ultrasound parameter to assess muscle mass (19), and GM thickness of <15 mm can be used to suggest a low muscle mass (18).

The prone position is the most common position while performing the ultrasonic examination of the posterior muscles of the lower limbs (including the GM) because this position allows better observation of the longitudinal and transverse muscle planes (16, 18, 20–22). However, in clinical practice, we have observed that a few older adults with disability fail to complete the examination in the prone position, and hence, in such cases, the sonographer has to perform the examination with patients acquiring other postures. The effect of patients acquiring these non-prone positions during ultrasonic examination on the diagnosis of the low muscle mass remains unknown.

This study aimed to examine the accuracy and consistency of different ultrasound measurement protocols (method A, prone position; method B, lateral position with legs separated; and method C, lateral position with legs together) repeated two or three times to identify a low muscle mass in men and women. This study was directed toward identifying a suitable measurement scheme of ultrasonic measurement of the muscle mass that can be more feasible for older adults with disability.

## Methods

### Study design and patients’ characteristics

This cross-sectional study was conducted from May 9, 2023, to May 29, 2023 and included patients who were admitted to our hospital and participants from nursing homes. Other inclusion criteria were patients who were ≥ 60 years and could undergo prone and lateral position during the ultrasound examination. Patients were excluded based on the following exclusion criteria: (1) localized skin infection, (2) inability to cooperate, (3) combined mental disorder, (4) hemiplegia caused by stroke, and (5) examination refusal.

### Ethics statement

This study was conducted following the tenets of the Declaration of Helsinki and was approved by the Institutional Review Board (IRB) of a mental health center in western China (IRB number: 2021-06-01). Signed informed consent was obtained from all the participants or their legal guardians.

### Ultrasonic measurement

**Figure 1:**
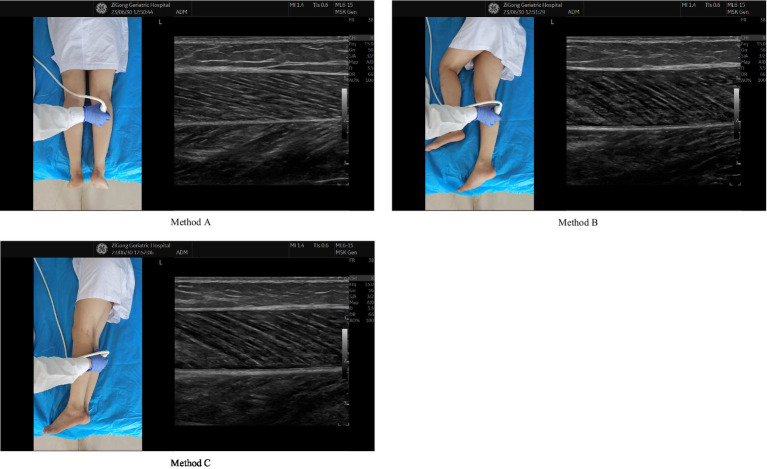
Three different sonographic protocols. Method A lying prone on the examination bed with both legs straight and relaxed and both feet hanging outside the examination bed. Method B lying on the right side with the left leg flexed and the right leg relaxed. Method C lying on the right side with legs together with the lower body muscles in a relaxed state.

Each participant underwent ultrasonic measurements in three different body postures, and for each body posture, three measurements were performed. The following are the three measurement schemes based on body posture (See Figure 1 for details): (1) method A, lying prone on the examination bed with both legs straight and relaxed and both feet hanging outside the examination bed (16, 17), (2) method B, lying on the right side with the left leg flexed and the right leg relaxed, and (3) method C, lying on the right side with legs together with the lower body muscles in a relaxed state. The inspector used the ultrasonic probe of the ultrasound diagnostic instrument (GE LOGIQ eNextGen Ultrasound Instrument, USA) to examine the muscle thickness close to the skin. The probe was placed on the thickest area of the medial head of the GM parallel to the long axis of the muscle, and the muscle thickness was measured (23). The thickness was measured as the distance between the superficial and deep fascia of the GM (23) and to the nearest 0.01 cm. Although the measurement was mainly performed on the right lower extremity, the left lower extremity was measured in cases with wound on the right lower extremity or a recent fracture of the right lower extremity, provided that the lower extremities were symmetrical. After performing three consecutive measurements for each posture, the average of two and three measurements of the GM thickness was taken (18, 23). All ultrasound measurements were performed independently by a senior sonographer (with >20 years of experience). A low muscle mass is defined as a GM thickness of <15 mm (18).

### Covariates

Covariate data, which were collected using face-to-face interviews, included age, hypertension, diabetes, coronary heart disease (CHD), chronic obstructive pulmonary disease (COPD), and activity of daily living (ADL) score.

### Statistical analyses

All the statistical analysis was performed using SPSS 23.0 (IBM Corp, NY, United States). Continuous data with normal and non-normal distributions are presented as mean ± standard deviation (SD) and median and interquartile range (IQR), respectively, whereas categorical data are represented as quantity (%). The level of significant was set at a *p* value of <0.05, with all tests being two-sided. Baseline characteristics were compared using Student’s *t*, rank-sum, and Pearson chi-square tests. Data of three repeated measurements performed using the method A were used as the reference to estimate the receiver operating characteristic (ROC) curve and determine the accuracy of other measurement protocols in identifying the low muscle mass. Area under the curve (AUC), specificity, sensitivity, positive predictive value (PPV), negative predictive value (NPV), and Kappa values were also measured.

## Results

This study included 489 individuals, of whom 221 (45.19%) were male. Table 1 demonstrates the baseline characteristics of all included participants. Women with age > 80 years were more in number, and a greater number of men had a high blood pressure, COPD, and disability (Table 1). The mean GM thickness was higher in men compared with that in women, regardless of the measurement protocol (Table 1).

**Table 1:** Baseline characteristics of participants according to the sex.

Characteristics	Male (*n* = 221)	Female (*n* = 268)	*p*-value
Age, years, *n* (%)			0.01
<80	168(49)	175(51)	
≥80	53(36.3)	93(63.7)	
Hypertension, *n* (%)			0.042
No	121(41.4)	171(58.6)	
Yes	100(50.8)	97(49.2)	
Diabetes, *n* (%)			0.957
No	181(45.1)	220(54.9)	
Yes	40(45.5)	48(54.5)	
CHD, *n* (%)			0.13
No	171(43.5)	222(56.5)	
Yes	50(52.1)	46(47.9)	
COPD, *n* (%)			<0.001
No	172(39.9)	259(60.1)	
Yes	49(84.5)	9(15.5)	
ADL score, *n* (%)			0.043
100	116(41.3)	165(58.7)	
<100	105(50.5)	103(49.5)	
Gastrocnemius muscle thickness, mm, mean (SD)			
2(A)	16.37(2.58)	15.63(2.61)	0.002
3(A)	16.41(2.6)	15.66(2.62)	0.002
2(B)	16.16(2.56)	15.35(2.6)	0.001
3(B)	16.17(2.55)	15.37(2.6)	0.001
2(C)	16.01(2.59)	15.26(2.66)	0.002
3(C)	16.03(2.6)	15.25(2.66)	0.001

A represents the measurement method in the prone position.

B represents the measurement method in lateral position with legs apart.

C represents the measurement method in the lateral position with the legs together.

2 refers to taking the average of 2 measurement results. 3 refers to the average value of 3 measurements.

CHD: coronary heart disease; COPD: chronic obstructive pulmonary disease; ADL: activities of daily living.

The difference in the prevalence of low muscle mass identified between two and three replicates of the same measurement protocol ranged from 0 to 1.3% (Table 2). Among males, three repeated measurements of method C and A identified the highest (35.7%) and lowest (28.5%) prevalence of low muscle mass, respectively, whereas among females, two or three repeated measurements of method B and A identified the highest (41.8%) and lowest (34.7%) prevalence of low muscle mass, respectively (Table 2).

**Table 2:** Differences between males and females in different ultrasound measurement protocols.

Gastrocnemius muscle thickness, mm	Male (*n* = 221)	Female (*n* = 268)	*p*-value
**2(A)**			0.176
≥15	157(71)	175(65.3)	
<15	64(29)	93(34.7)	
**3(A)**			0.144
≥15	158(71.5)	175(65.3)	
<15	63(28.5)	93(34.7)	
**2(B)**			0.028
≥15	150(67.9)	156(58.2)	
<15	71(32.1)	112(41.8)	
**3(B)**			0.021
≥15	151(68.3)	156(58.2)	
<15	70(31.7)	112(41.8)	
**2(C)**			0.179
≥15	145(65.6)	160(59.7)	
<15	76(34.4)	108(40.3)	
**3(C)**			0.265
≥15	142(64.3)	159(59.3)	
<15	79(35.7)	109(40.7)	

A represents the measurement method in the prone position.

B represents the measurement method in lateral position with legs apart.

C represents the measurement method in the lateral position with the legs together.

2 refers to taking the average of 2 measurement results. 3 refers to the average value of 3 measurements.

When the three repeated measurements of the method A was set as the reference, the sensitivity, specificity, AUC, and Kappa values (from low to high) were 93.70–98.40%, 88–98.70%, 0.977–1, and 0.773, 0.801, 0.829, 0.839, and 0.967, respectively, for males and 93.50–98.90%, 87.40–99.40%, 0.973–1, and 0.786, 0.794, 0.804, 0.819, and 0.984, respectively, for females (Table 3).

**Table 3:** Accuracy and consistency of different gastrocnemius muscle thickness measurement protocols.

Postures	Cut-off	Sensitivity	Specificity	AUC	PPV	NPV	Kappa value
Male							
3A	15 mm	Ref.	Ref.	Ref.	Ref.	Ref.	Ref.
2A	15 mm	98.40%	98.70%	1(0.999–1)	0.97	0.99	0.967
2B	15 mm	93.70%	92.40%	0.988(0.979–0.998)	0.83	0.97	0.829
3B	15 mm	93.70%	93%	0.988(0.978–0.997)	0.84	0.97	0.839
2C	15 mm	95.20%	89.90%	0.978(0.963–0.993)	0.79	0.98	0.801
3C	15 mm	95.20%	88%	0.977(0.96–0.993)	0.76	0.98	0.773
Female							
3A	15 mm	Ref.	Ref.	Ref.	Ref.	Ref.	Ref.
2A	15 mm	98.90%	99.40%	1(0.999–1)	0.99	0.99	0.984
2B	15 mm	97.80%	88%	0.992(0.985–0.998)	0.81	0.99	0.819
3B	15 mm	96.80%	87.40%	0.991(0.983–0.998)	0.8	0.98	0.804
2C	15 mm	93.50%	88%	0.973(0.954–0.992)	0.81	0.96	0.786
3C	15 mm	94.60%	88%	0.973(0.954–0.992)	0.81	0.97	0.794

A represents the measurement method in the prone position.

B represents the measurement method in lateral position with legs apart.

C represents the measurement method in the lateral position with the legs together.

2 refers to taking the average of 2 measurement results. 3 refers to the average value of 3 measurements.

## Discussion

To our knowledge, this study, for the first time, demonstrated high accuracy and consistency of different ultrasound protocols in identifying the low muscle mass in individuals with age > 60 years. The results presented in this study will be useful in clinical situations to manage cases where older adults with disability cannot complete the analysis of the muscle mass due to stiff and flexed joints. This study revealed that, in such cases, different body postures may be allowed during the ultrasound examination without compromising the effectiveness of detecting the low muscle mass.

In previous studies, the prevalence of the low muscle mass in males and females was reported to be 17–85% and 17–68%, respectively (24–26). Consistent with these results, the prevalence of the low muscle mass in males and females was found to be 28.5–35.7% and 34.7–41.8%, respectively. This data suggest that the prevalence of the low muscle mass in older adults (> 60 years) is high, and hence targeted screening and timely intervention are needed to minimize the occurrence of disabling comorbid sarcopenia.

Among different ultrasound protocols, method A is the most commonly used, and hence in the present study we used it as a reference method. Method B can be completed by most of the individuals with disability; however, a few older adults with limited leg separation, method C was selected during the measurement. In method C, the left limb may squeeze the muscles of the right limb, thereby affecting the accuracy of the measurement. In both males and females, method A demonstrated the lowest prevalence of the low muscle mass, whereas both methods B and C identified a slightly higher prevalence of the low muscle mass. Based on the result of the present study, we recommend the use of method B for patients who are not able to complete the evaluation using the method A, and in cases where both methods A and B are not feasible, the method C should be employed to identify the low muscle mass.

In previous studies, during the ultrasound evaluation of the GM thickness, measurements were repeated two (18, 27, 28) or three (23) times to achieve better accuracy. In the present study, we also compared the difference between measurements repeated two and three times to identify the low muscle mass. A small difference in the prevalence of the low muscle mass was found between measurements repeated two and three times (0–0.4% in women and slightly larger difference in men). This marginal difference may be related to the differences in the structure and thickness of the GM between men and women (29, 30). We also found that the accuracy and consistency of the muscle mass measurement repeated two times were also high, and hence it is possible to identify the low muscle mass by repeating the ultrasound measurement either two or three times.

This study had a few limitations. First, the number of participants was less. In addition, due to the presence of disabilities in the participants, nearly half were unable to complete height or weight measurements. Therefore, it was not possible to analyze the relationship between BMI and muscle mass. Second, the muscle thickness was not measured in sitting position. Although a few studies have conducted measurements in the sitting position (31), our aim was to determine feasible measurement conditions for older adults with disability, and hence we did not measure the muscle thickness in the sitting position. Third, as there is currently no consensus or guidelines on the protocol for ultrasound measurement of GM thickness, we could only refer to the protocols for ultrasound measurement of GM thickness in the published literature for use as the standard protocol in our study. Our next step will be to improve DEXA as the gold standard to delineate cutoff values for in-depth research. Finally, in the study, only the thickness of GM was measured. The thickness of other muscles, such as rectus femoris, vastus lateralis, tibialis anterior (32), was not measured, and further studies will be required in the future to explore this aspect.

## Conclusion

All the different ultrasound protocols led to high accuracy and consistency in identifying a low muscle mass. We suggest that if method A cannot be used in an older adult with disability, method B should be employed for measuring the GM thickness. Repeating the measurements two or three times was found to be feasible.

## Data availability statement

The datasets generated and analyzed during the current study are not publicly available since it is part of a cohort study. The datasets will be available 2 years after publication and are currently available from the corresponding author on a reasonable request.

## Ethics statement

The studies involving humans were approved by Research Ethics Committee of Zigong Affiliated Hospital of Southwest Medical University, Zigong Mental Health Center (No.2021-06-01). The studies were conducted in accordance with the local legislation and institutional requirements. The participants provided their written informed consent to participate in this study.

## Author contributions

HD: Conceptualization, Data curation, Formal analysis, Investigation, Methodology, Writing – original draft. XL: Writing – original draft, Conceptualization, Data curation, Formal analysis, Investigation, Methodology. SH: Conceptualization, Data curation, Formal analysis, Investigation, Methodology, Writing – original draft. JL: Conceptualization, Data curation, Formal analysis, Investigation, Methodology, Writing – review & editing. ZL: Conceptualization, Data curation, Formal analysis, Investigation, Methodology, Writing – review & editing. LC: Conceptualization, Data curation, Formal analysis, Investigation, Methodology, Writing – review & editing. LZ: Conceptualization, Data curation, Formal analysis, Investigation, Methodology, Writing – review & editing. YX: Conceptualization, Data curation, Formal analysis, Investigation, Methodology, Writing – review & editing. QN: Conceptualization, Data curation, Formal analysis, Investigation, Methodology, Writing – review & editing. XC: Conceptualization, Data curation, Formal analysis, Funding acquisition, Investigation, Methodology, Writing – review & editing.

## References

[1] Qiao R Jia S Zhao W Xia X Su Q Hou L. Prevalence and correlates of disability among urban-rural older adults in Southwest China: a large, population-based study. BMC Geriatr. (2022) 22:517. (In eng). doi: 10.1186/s12877-022-03193-2, PMID: 35739469 PMC9229854

[2] Zheng PP Guo ZL Du XJ Yang HM, Wang ZJ. Prevalence of disability among the Chinese older population: a systematic review and Meta-analysis. Int J Environ Res Public Health (2022) 19:1656. (In eng). doi: 10.3390/ijerph19031656, PMID: 35162679 PMC8835133

[3] Yau PN Foo CJE Cheah NLJ Tang KF Lee SWH. The prevalence of functional disability and its impact on older adults in the ASEAN region: a systematic review and meta-analysis. Epidemiol. Health. (2022) 44:e2022058. (In eng). doi: 10.4178/epih.e2022058, PMID: 35843601 PMC9754909

[4] Okoro CA Hollis ND Cyrus AC Griffin-Blake S. Prevalence of disabilities and health care access by disability status and type among adults - United States, 2016. MMWR Morb Mortal Wkly Rep. (2018) 67:882–7. (In eng). doi: 10.15585/mmwr.mm6732a3, PMID: 30114005 PMC6095650

[5] Hosseinpoor AR Bergen N Kostanjsek N Kowal P Officer A Chatterji S. Socio-demographic patterns of disability among older adult populations of low-income and middle-income countries: results from world health survey. Int J Public Health. (2016) 61:337–45. (In eng). doi: 10.1007/s00038-015-0742-3, PMID: 26537634 PMC4879166

[6] Zeng Y Feng Q Hesketh T Christensen K Vaupel JW. Survival, disabilities in activities of daily living, and physical and cognitive functioning among the oldest-old in China: a cohort study. Lancet (London, England). (2017) 389:1619–29. (In eng). doi: 10.1016/S0140-6736(17)30548-2, PMID: 28285816 PMC5406246

[7] Oveisgharan S Yu L Bennett DA Buchman AS. Incident mobility disability, parkinsonism, and mortality in community-dwelling older adults. PLoS One. (2021) 16:e0246206. (In eng). doi: 10.1371/journal.pone.0246206, PMID: 33534811 PMC7857621

[8] Pongiglione B De Stavola BL Kuper H Ploubidis GB. Disability and all-cause mortality in the older population: evidence from the English longitudinal study of ageing. Eur J Epidemiol. (2016) 31:735–46. doi: 10.1007/s10654-016-0160-8, PMID: 27177908 PMC5005412

[9] Fried TR Bradley EH Williams CS Tinetti ME. Functional disability and health care expenditures for older persons. Arch Intern Med. (2001) 161:2602–7. (In eng). doi: 10.1001/archinte.161.21.2602 11718592

[10] Kitamura A Seino S Abe T Nofuji Y Yokoyama Y Amano H. Sarcopenia: prevalence, associated factors, and the risk of mortality and disability in Japanese older adults. J Cachexia Sarcopenia Muscle. (2021) 12:30–8. (In eng). doi: 10.1002/jcsm.12651, PMID: 33241660 PMC7890144

[11] An HJ Tizaoui K Terrazzino S Cargnin S Lee KH Nam SW. Sarcopenia in autoimmune and rheumatic diseases: a comprehensive review. Int J Mol Sci. (2020) 21:21(16) (In eng). doi: 10.3390/ijms21165678, PMID: 32784808 PMC7461030

[12] Tsekoura M Kastrinis A Katsoulaki M Billis E Gliatis J. Sarcopenia and its impact on quality of life. Adv Exp Med Biol. (2017) 987:213–8. (In eng). doi: 10.1007/978-3-319-57379-3_19 28971460

[13] Cruz-Jentoft AJ Sayer AA. Sarcopenia. Lancet (London, England). (2019) 393:2636–46. (In eng). doi: 10.1016/S0140-6736(19)31138-9 31171417

[14] Chen L-K Woo J Assantachai P Auyeung TW Chou MY Iijima K. Consensus update on sarcopenia diagnosis and treatment. J Am Med Dir Assoc. (2019) 21:300 (In eng)–307.e2. doi: 10.1016/j.jamda.2019.12.012, PMID: 32033882

[15] Yuguchi S Asahi R Kamo T Azami M Ogihara H. Gastrocnemius thickness by ultrasonography indicates the low skeletal muscle mass in Japanese elderly people. Arch Gerontol Geriatr. (2020) 90:104093. (In eng). doi: 10.1016/j.archger.2020.104093, PMID: 32526562

[16] Barotsis N Galata A Hadjiconstanti A Panayiotakis G. The ultrasonographic measurement of muscle thickness in sarcopenia. A prediction study. Eur J Phys Rehabil Med. (2020) 56:427–37. (In eng). doi: 10.23736/S1973-9087.20.06222-X, PMID: 32293812

[17] Kuyumcu ME Halil M Kara Ö Çuni B Çağlayan G Güven S. Ultrasonographic evaluation of the calf muscle mass and architecture in elderly patients with and without sarcopenia. Arch Gerontol Geriatr. (2016) 65:218–24. (In eng). doi: 10.1016/j.archger.2016.04.004, PMID: 27107379

[18] Wang J Hu Y Tian G. Ultrasound measurements of gastrocnemius muscle thickness in older people with sarcopenia. Clin Interv Aging. (2018) 13:2193–9. (In eng). doi: 10.2147/CIA.S179445, PMID: 30464428 PMC6214412

[19] Zhao R Li X Jiang Y Su N Li J Kang L. Evaluation of appendicular muscle mass in sarcopenia in older adults using ultrasonography: a systematic review and Meta-analysis. Gerontology. (2022) 68:1174–98. (In eng). doi: 10.1159/000525758, PMID: 35878591 PMC9677864

[20] Berrigan WA Wickstrom J Farrell M Alter K. Hip position influences shear wave elastography measurements of the hamstring muscles in healthy subjects. J Biomech. (2020) 109:109930. (In eng). doi: 10.1016/j.jbiomech.2020.109930, PMID: 32807303 PMC7486790

[21] Hirata K Yamadera R Akagi R. Associations between range of motion and tissue stiffness in young and older people. Med Sci Sports Exerc. (2020) 52:2179–88. (In eng). doi: 10.1249/MSS.0000000000002360, PMID: 32348099 PMC7497479

[22] Chen CP Tang SF Hsu CC Chen RL Hsu RC Wu CW. A novel approach to sonographic examination in a patient with a calf muscle tear: a case report. J Med Case Rep. (2009) 3:7291. (In eng). doi: 10.4076/1752-1947-3-7291, PMID: 19830167 PMC2726513

[23] Wang S Zhang Y Wang C. Clinical application of multimodal ultrasound on muscle mass and stiffness in patients with sarcopenia (in Chinese). Chinese Journal of Geriatrics. (2022) 41:534–8. doi: 10.3760/cma.j.issn.0254-9026.2022.05.006

[24] Wong L Kent AB Lee D Roberts MA McMahon LP. Low muscle mass and early hospital readmission post-kidney transplantation. Int Urol Nephrol. (2022) 54:1977–86. (In eng). doi: 10.1007/s11255-021-03085-1, PMID: 35028810

[25] Tey SL Huynh DTT Berde Y Baggs G How CH Low YL. Prevalence of low muscle mass and associated factors in community-dwelling older adults in Singapore. Sci Rep. (2021) 11:23071. (In eng). doi: 10.1038/s41598-021-02274-3, PMID: 34845250 PMC8630119

[26] Graf CE Pichard C Herrmann FR Sieber CC Zekry D Genton L. Prevalence of low muscle mass according to body mass index in older adults. Nutrition. (2017) 34:124–9. (In eng). doi: 10.1016/j.nut.2016.10.002 28063507

[27] Raj IS Bird SR Shield AJ. Reliability of ultrasonographic measurement of the architecture of the vastus lateralis and gastrocnemius medialis muscles in older adults. Clin Physiol Funct Imaging. (2012) 32:65–70. (In eng). doi: 10.1111/j.1475-097X.2011.01056.x, PMID: 22152081

[28] Isaka M Sugimoto K Yasunobe Y Akasaka H Fujimoto T Kurinami H. The usefulness of an alternative diagnostic method for sarcopenia using thickness and Echo intensity of lower leg muscles in older males. J Am Med Dir Assoc. (2019) 20:1185.e1–8. (In eng). doi: 10.1016/j.jamda.2019.01.152, PMID: 30902675

[29] Fujiwara K Asai H Toyama H Kunita K Yaguchi C Kiyota N. Changes in muscle thickness of gastrocnemius and soleus associated with age and sex. Aging Clin Exp Res. (2010) 22:24–30. (In eng). doi: 10.1007/BF03324811 19920407

[30] Chow RS Medri MK Martin DC Leekam RN Agur AM McKee NH. Sonographic studies of human soleus and gastrocnemius muscle architecture: gender variability. Eur J Appl Physiol. (2000) 82:236–44. (In eng). doi: 10.1007/s004210050677, PMID: 10929218

[31] Fukumoto Y Ikezoe T Taniguchi M Yamada Y Sawano S Minani S. Cut-off values for lower limb muscle thickness to detect Low muscle mass for sarcopenia in older adults. Clin Interv Aging. (2021) 16:1215–22. (In eng). doi: 10.2147/CIA.S304972, PMID: 34211270 PMC8241812

[32] Minetto MA Caresio C Menapace T Hajdarevic A Marchini A Molinari F. Ultrasound-based detection of Low muscle mass for diagnosis of sarcopenia in older adults. PM R. (2016) 8:453–62. (In eng). doi: 10.1016/j.pmrj.2015.09.014, PMID: 26431809

